# A multispecies outbreak of carbapenem-resistant bacteria harboring the *bla*_KPC_ gene in a non-classical transposon element

**DOI:** 10.1186/s12866-021-02169-3

**Published:** 2021-04-09

**Authors:** Aniela Wozniak, Cristian Figueroa, Francisco Moya-Flores, Piero Guggiana, Claudia Castillo, Lina Rivas, José M. Munita, Patricia C. García

**Affiliations:** 1 Laboratory of Microbiology, Department of Clinical Laboratories, Centro Médico San Joaquín, Escuela de Medicina, Pontificia Universidad Católica de Chile, 3rd floor, Vicuña Mackenna, 4686 Santiago, Chile; 2 Clinical Laboratories Network, Red de Salud UC-CHRISTUS, Santiago, Chile; 3 Millennium Initiative for Collaborative Research On Bacterial Resistance (MICROB-R), Santiago, Chile; 4 Genomics & Resistant Microbes group (GeRM), Facultad de Medicina Clinica Alemana, Universidad del Desarrollo, Santiago, Chile

**Keywords:** KPC Carbapenemase, Carbapenem resistant *Enterobacteriaceae*, Multispecies outbreak, Horizontal gene transfer

## Abstract

**Background:**

*Klebsiella pneumoniae* is the most frequent KPC-producing bacteria. The *bla*_KPC_ gene is frequently embedded in Tn4401 transposon, and less frequently in non-Tn4401 elements (NTE_KPC_) variants I-III. The first case of KPC in the UC-CHRISTUS Clinical Hospital was detected in *Pseudomonas aeruginosa*. Soon after this event, KPC was detected in 2 additional *Pseudomonas aeruginosa*, 3 *Escherichia coli*, 3 *Enterobacter cloacae*, 3 *Klebsiella pneumoniae,* and 1 *Citrobacter freundii*, isolated from 6 different patients. We aimed to elucidate the possible mechanisms of genetic transfer and dissemination of the *bla*_KPC_ gene among isolates of this multispecies outbreak. A molecular epidemiology analysis of the above mentioned clinical isolates (*n* = 13) through Multi-Locus Sequence Typing, plasmid analysis, Pulsed-Field Gel-Electrophoresis, and Whole-genome sequencing (WGS) was performed.

**Results:**

High-risk sequence types were found: *K. pneumoniae* ST11, *P. aeruginosa* ST654, and *E. cloacae* ST114. All enterobacterial isolates were not clonal except for 3 *E. coli* isolated from the same patient. WGS analysis in 6 enterobacterial isolates showed that 4 of them had *bla*_KPC_ embedded in a novel variant of NTE_KPC_ designated NTE_KPC_-IIe. Upstream of *bla*_KPC_ gene there was a 570 pb truncated *bla*_TEM-1_ gene followed by an insertion sequence that was 84% similar to ISEc63, a 4473 bp element of the Tn3 family. Downstream the *bla*_KPC_ gene there was a truncated ISKpn6 gene, and the inverted repeat right sequence of Tn4401. The ISec63-like element together with the *bla*_KPC_ gene plus Tn4401 remnants were inserted in the Tra operon involved in conjugative transfer of the plasmid. This NTE was carried in a broad host-range IncN plasmid. *P. aeruginosa* isolates carried *bla*_KPC_ gene embedded in a typical Tn4401b transposon in a different plasmid, suggesting that there was no plasmid transfer between *Enterobacteriaceae* and *P. aeruginosa* as initially hypothesized.

**Conclusions:**

Most enterobacterial isolates had *bla*_KPC_ embedded in the same NTE_KPC_-IIe element, suggesting that this multispecies KPC outbreak was due to horizontal gene transfer rather than clonal spread. This poses a greater challenge to infection control measures often directed against containment of clonal spread.

**Supplementary Information:**

The online version contains supplementary material available at 10.1186/s12866-021-02169-3.

## Background

Carbapenem-resistant bacteria are a serious public health threat worldwide. Carbapenems are used as last resort antibiotics in infections caused by multidrug-resistant bacteria and carbapenemases are threatening this valuable therapeutic agent [1]. KPC is the most clinically significant class A carbapenemase because KPC-producing bacteria are susceptible to only a few antibiotics (colistin, aminoglycosides, tigecycline, and ceftazidime/avibactam) and patients infected with them have poor outcomes [2]. KPC carbapenemase was first reported in the United States in 1996 in a *Klebsiella pneumoniae* isolate [3] and a few years later it became endemic in regions like United States, Italy, Israel and Colombia [1]. To date, there are more than 30 KPC variants described, with KPC-2 and KPC-3 being the most frequently encountered [4]. *K. pneumoniae* is by far the most frequent species carrying *bla*_KPC_. However, it has been described in several other species of *Enterobacteriaceae* [5]. Albeit less frequently, KPC-producing *Pseudomonas aeruginosa* has also been described [6, 7].

The worldwide dissemination of KPC-producing *K. pneumoniae* has been associated with the successful spread of a specific genetic lineage designated clonal group 258 (CG258). This CG contains 43 different sequence types (STs), with ST258, and ST512 being the predominant ones. Indeed, ST258 is a high-risk clone responsible for 80% of KPC-producing *K. pneumoniae* outbreaks in the United States [8]. However, other mechanisms of KPC dissemination have been described, for example horizontal transfer of mobile genetic elements [9]. The KPC-coding gene, *bla*_KPC_, is usually found within a Tn*4401* transposon, a mobile genetic element derived from the Tn3 transposon family that facilitates its spread [8, 10]. However, the *bla*_KPC_ gene has also been found in non Tn4401 elements (NTE). The first NTE was described in China in 2007 [11] and additional NTEs with different structures were later described in countries like Argentina [12], Colombia [9], Chile [13], and Brazil [14–16]. The *bla*_KPC_ gene is usually carried on plasmids of different incompatibility groups (Inc). A recent meta-analysis made with 435 KPC-bearing plasmids, showed that the most frequent incompatibilty group was IncN [4]. These elements have contributed to the dissemination of KPC within *K. pneumoniae* and other bacterial species.

The first KPC-producing strain in Chile was detected in 2012 in a *K. pneumoniae* isolated from a patient traveling from Italy [17]. Since then, KPC has been found in several species of *Enterobacteriaceae* throughout the country [13]*.* Surprisingly, the first KPC-producing strain in our University Hospital (Clinical Hospital of Red de Salud UC-CHRISTUS) was detected in 2015 in a *P. aeruginosa* strain recovered from an adult patient at the intensive care unit. Shortly after the detection of this first KPC-producing *P. aeruginosa*, KPC was subsequently found in several species of *Enterobacteriaceae* in various hospital units, in a relatively short period (2 months) [7]; the event fulfilled the classical outbreak definition of The World Health Organization [18]. This observation led us to hypothesize that the *bla*_KPC_ gene might have been transferred from the index KPC-*P. aeruginosa* to *Enterobacteriaceae* through horizontal gene transfer. Therefore, we aimed to elucidate the possible mobile genetic elements harboring KPC and possible mechanisms of genetic transfer and dissemination of the *bla*_*KPC*_ gene among bacterial isolates associated with this multi-species outbreak.

## Results

### Isolates included in the outbreak analysis

A detailed molecular analysis of 3 *P. aeruginosa* and 10 *Enterobacteriaceae* associated with the outbreak, obtained from six different patients was performed. The index KPC-*P. aeruginosa* (Pae-1) was isolated from P1 in the ICU (Table 1) and it was also found to carry *bla*_VIM_ (Table 2). The first KPC-producing *K. pneumoniae* (Kpn-3) was isolated almost 20 days later from P3 in the step-down unit. One month later, three KPC-producing isolates were obtained from the same patient (P3) in the same unit: *K. pneumoniae* (Kpn-4), *E. coli* (Eco-5) and *E. cloacae* (Ecl-6). Eleven days later three additional KPC-producing *E. coli* (Eco-7, Eco-8) and one *E. cloacae* (Ecl-9) were again recovered from P3. At the same time of isolation of the first KPC-producing *K. pneumoniae*, a new KPC-producing *K. pneumoniae* (Kpn-10) was recovered from P4 in the surgical unit, and two KPC-producing isolates of *C. freundii* (Cfr-11) and one *E. cloacae* (Ecl-12) respectively, were recovered from P5 in the pediatric ICU. Of note, the two isolates from the pediatric care unit also carried the *bla*_VIM_ gene (Table 2). Two additional *P. aeruginosa* isolates were included: one of them was the following consecutive VIM-positive *P. aeruginosa* (Pae-2) isolated from P2 in the emergency department 24 days after the index case, and the second was Pae-13, isolated more than 2 months after the index case in the same ICU, but from a different patient (P6) (Tables 1 and 2). All isolates carried *bla*_KPC-2_, except for Pae-2, that only harbored *bla*_VIM_. All *P. aeruginosa* isolates carried the bla_VIM-2_ metallo-beta-lactamase. In contrast, Cfr-11 and Ecl-12 carried *bla*_VIM-1_ (Table 2).

**Table 1:** Isolates included in this study

Patient N°	Isolate	Date of isolation	Hospital Unit	Species	Selection criteria for inclusion in the study
1	Pae-1	05-05-2015	ICU	*P. aeruginosa*	Index case: 1st *bla*_KPC_ isolate in the hospital (KPC + VIM)
2	Pae-2	29-05-2015	Emergency	*P. aeruginosa*	2nd consecutive case of VIM-positive *P. aeruginosa*
3	**Kpn-3**	01-06-2015	Step-down	*K. pneumoniae*	1st KPC-positive *K. pneumoniae* in the hospital
	Kpn-4	09-07-2015	Step-down	*K. pneumoniae*	1st case of a patient colonized with 3 different bacterial species coding for KPC
	**Eco-5**			*E. coli*	
	**Ecl-6**			*E. cloacae*	
	**Eco-7**	20-07-2015	Coronary care	*E. coli*	3rd Surveillance of the same patient again with KPC-positive bacteria
	**Eco-8**			*E. coli*	
	**Ecl-9**			*E. cloacae*	
4	Kpn-10	04-06-2015	Surgical	*K. pneumoniae*	2nd *K. pneumoniae* KPC-positive in the hospital
5	Cfr-11	04-06-2015	Pediatrics	*C. freundii*	1st case of KPC in the Pediatric Unit
	Ecl-12			*E. cloacae*	
6	**Pae-13**	18-07-2015	ICU	*P. aeruginosa*	Similar to index case

Isolates analyzed through WGS are shown in boldface letters

**Table 2:** Sequence types, plasmids, and pulsotypes determined in this study

Patient N°	Isolate	Carbapenemase	Pulsotype (PFGE)	MLST	Plasmid incompatibility group	Genetic environment of *bla*_KPC_
1	Pae-1	KPC-2 / VIM-2	P1	ST654	NF	Tn4401b
2	Pae-2	VIM-2	P2	ST282	NF	NA
3	Kpn-3	KPC-2	K1	ST11	IncN	NTE_KPC_-IIe
	Kpn-4	KPC-2	K2	ST11	IncN; IncA/C	ND
	Eco-5	KPC-2	E1	ST378	IncN; IncA/C	NTE_KPC_-IIe
	Ecl-6	KPC-2	L1	ST45	IncN; IncFIA; IncFIB; IncA/C	NTE_KPC_-IIe
	Eco-7	KPC-2	E2	ST378	IncN; IncA/C	NTE_KPC_-IIe
	Eco-8	KPC-2	E2	ST378	IncN; IncA/C	NTE_KPC_-IIe
	Ecl-9	KPC-2	L2	ST45	IncN; IncA/C	NTE_KPC_-IIe
4	Kpn-10	KPC-2	K3	ST25	IncN	ND
5	Cfr-11	KPC-2 / VIM-1	NA	ST130	IncN	ND
	Ecl-12	KPC-2 / VIM-1	L3	ST114	NF	ND
6	Pae-13	KPC-2 / VIM-2	P1	ST654	NF	Tn4401b

*NTE*_*KPC*_*-IIe* Non Tn4401 element variant IId, *NA* Not applicable, *ND* Not determined, *NF* Not found

### Molecular epidemiology of the isolates

**Fig. 1:**
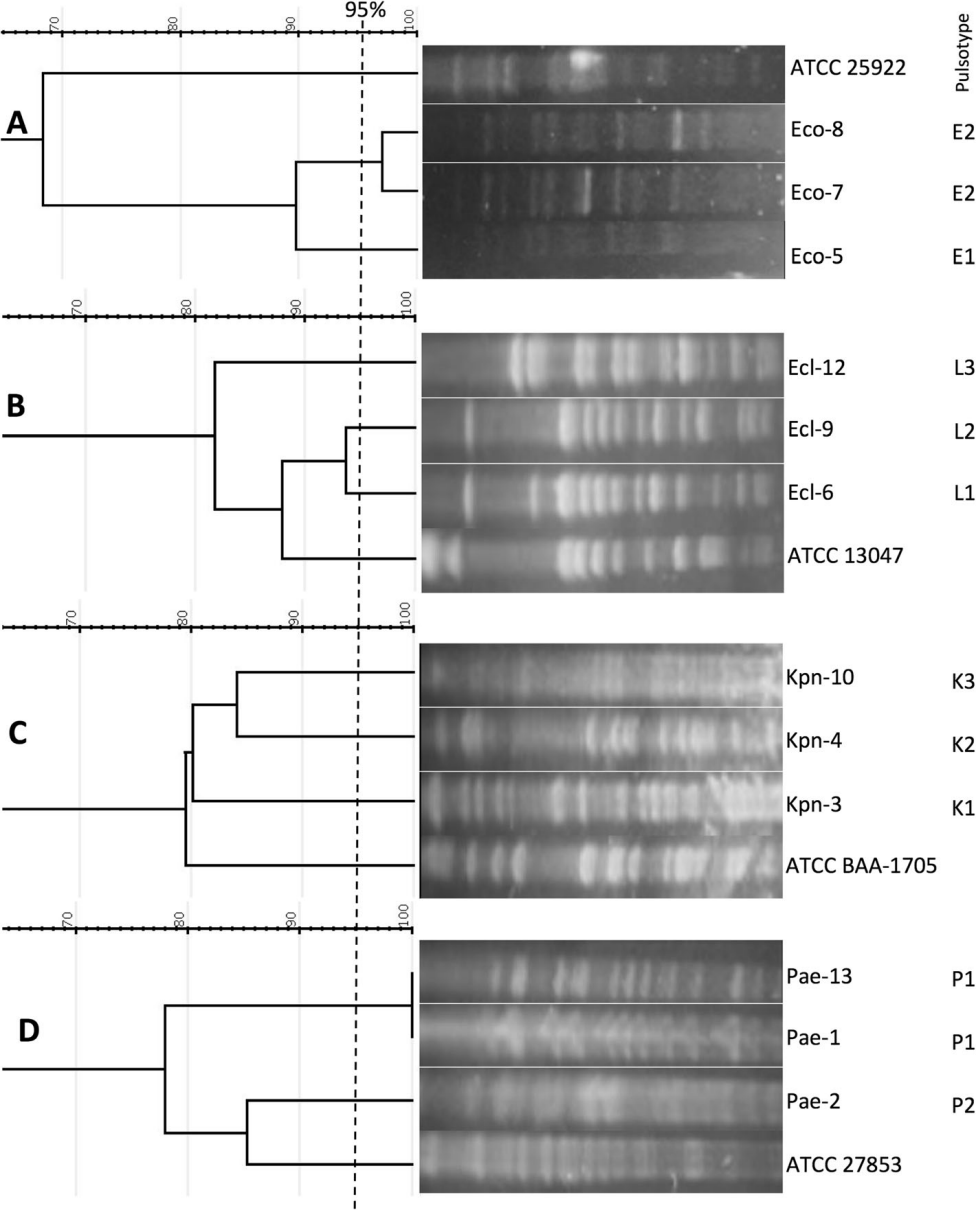
Dendrograms obtained from the analysis of PFGE patterns. Panels correspond to *E. coli* (**a**), *E. cloacae* (**b**), *K. pneumoniae* (**c**), and *P. aeruginosa* (**d**) isolates. Dendrograms were constructed using the Dice coefficient and unweighted pair group method with arithmetic mean (UPGMA). The 95% similarity cut-off is indicated with a dashed line. A standard ATCC strain of each species was included in every analysis. Pulsotypes for each isolate are indicated in the right column. Lanes that were non-adjacent in the original gel were cropped to be positioned according to dendrogram order. The unprocessed gel photographs are shown in Supplementary Fig. S1

PFGE analysis determined that isolates Pae-1 and Pae-13 were clonal (Fig. 1). Of note, those isolates were recovered from different patients and time points, but within the same ICU. All *E. coli* isolates were recovered from the same patient (P3), but only Eco-7 and Eco-8 were clonal. Eco-5, obtained 11 days before in a different hospital unit was a different strain. Similar to the case with Eco-5, *E. cloacae* isolates were not clonally related, despite two of them were recovered from the same patient (P3) within 11 days (Ecl-6 and Ecl-9), but in two different units. *K. pneumoniae* isolates were not clonal despite two of them, Kpn-3 and Kpn-4 were recovered from the same patient (P3). The unprocessed gel photographs are shown in Supplementary Fig. S1.

In terms of our MLST results, all three *E. coli* belonged to the ST378. *K. pneumoniae* isolates were ST11 (Kpn-3 and Kpn-4) and ST25 (Kpn-10). Both *E. cloacae* recovered from P3 were ST45, whereas Ecl-12 was ST114. The KPC-harboring *P. aeruginosa* isolates isolated from the ICU were ST654 and Pae-2 was ST282 (Table 2).

### Plasmid analysis

**Fig. 2:**
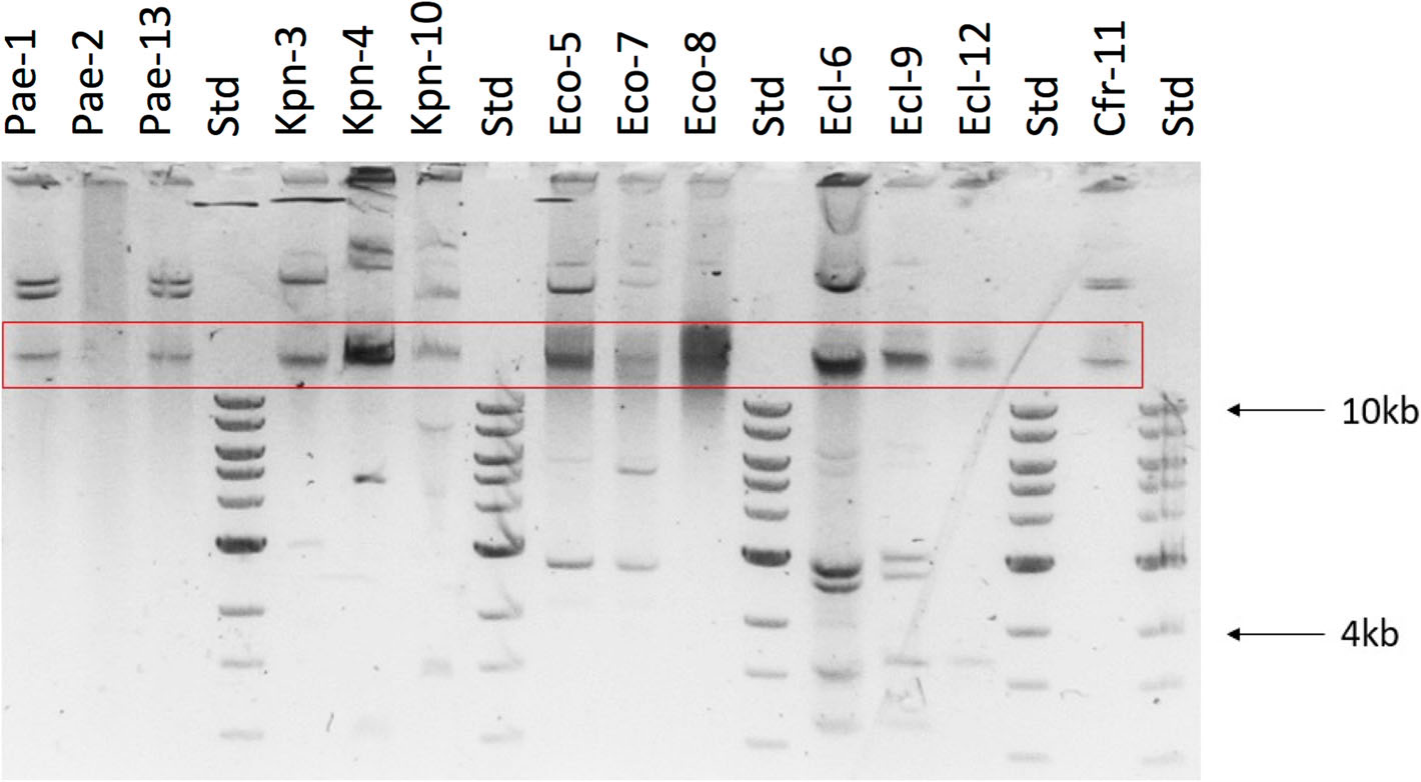
Agarose gel (0,75%) electrophoresis of plasmids extracted through alkaline lysis from the 13 isolates analyzed. Std: Molecular weight marker; the weight of the 10 kb and 4 kb bands are shown as a reference. The red box indicates the band corresponding to residual genomic DNA

*P. aeruginosa* isolates (Pae-1, Pae-2 and Pae-13) did not carry plasmids of any of the incompatibility groups most frequently found among *Pseudomonas* species, that were sought through PCR [19]. All but one (Ecl-12) enterobacterial isolates carried an IncN type plasmid (Table 2). Also, all isolates recovered from P3 except for the first *K. pneumoniae* isolate Kpn-3, carried a plasmid of the IncA/C type. The *E. cloacae* isolate Ecl-6 recovered from this patient additionally carried plasmids of the IncF1A and IncF1B types. Plasmids were extracted and visualized in a 0.75% agarose gel (Fig. 2). Typical patterns of relaxed, supercoiled and linear plasmid forms were observed in most isolates. Isolate Ecl-6 carried several plasmids, in accordance to PCR results. Although no plasmids were amplified in isolate Ecl-12 through PCR using a set of primers directed to the plasmids usually found in *Enterobacteriaceae*, it exhibited a band of less than 4 Kb (Fig. 2).

### Genetic environment of *bla*_KPC_ gene and plasmid

WGS was performed in 7/13 isolates, including KPC-*P. aeruginosa* Pae-13 and 6 isolates from P3: Kpn-3, Ecl-9, Eco-8, Eco-5, Eco-7, and Ecl-6.

**Fig. 3:**
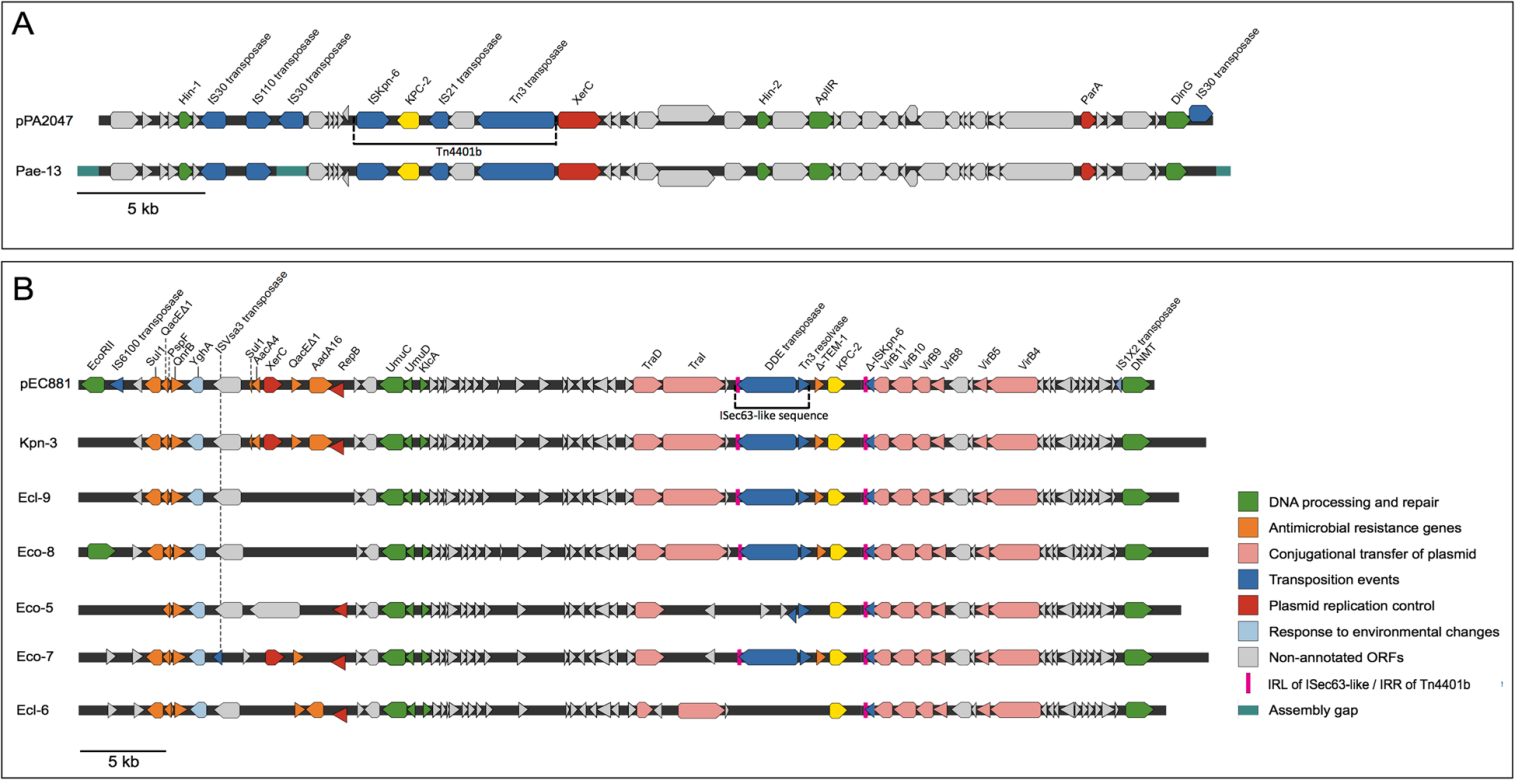
Genetic environment of *bla*_KPC_ gene in plasmids from *P. aeruginosa* (**a**) and enterobacterial isolates (**b**) obtained through WGS analysis. Plasmid from *P. aeruginosa* Pae-13 was obtained through assembly against plasmid pPA2047 and the *bla*_KPC_ gene embedded in Tn4401 is shown in yellow (**a**). Plasmids from enterobacterial isolates were obtained through assembly against plasmid pEC881_KPC and the *bla*_KPC_ gene is embedded in a NTE_KPC_-IIe element (**b**)

The *bla*_KPC_-containing contig of Pae-13 was 35,034 bp long (Genbank accession N° MT949191) and it aligned to a 43,660 bp plasmid (pPA2047) isolated in Argentina (Genbank accession N° MN082782, November 2019). Contigs were then assembled using plasmid pPA2047 as the reference sequence and the plasmid depicted in Fig. 3a was obtained. Analysis of the genetic environment showed that Pae-13 harbored *bla*_KPC_ embedded in an intact Tn*4401* transposon of the b isoform (Fig. 3a). This plasmid did not amplify any of the replicons corresponding to incompatibility groups of *Pseudomonas* species [19] or *Enterobacteriaceae* [20]. Moreover, it was non-typeable according to PlasmidFinder software [21].

The size of the *bla*_KPC_ gene-containing contigs in enterobacterial genomes ranged from 18,252 bp to 42,936 bp (Genbank accession N°: MT949189 for Kpn-3; MT949193 for Ecl-6, Ecl-9 and Eco-5; MT949190 for Eco-8; MT949192 for Eco-7). All of them aligned to a 59,373 bp long plasmid of the IncN type named pEC881_KPC recovered in 2013 from a carbapenem-resistant *E. coli* in Colombia (Genbank accession N° CP019026.1). Contigs were then assembled using plasmid pEC881_KPC as the reference sequence. The assembly retrieved the plasmid sequence with different identity and completeness levels for all six enterobacterial isolates analyzed (Fig. 3b). The *bla*_KPC_ gene was not embedded in transposon Tn4401 in any of the analyzed enterobacterial genomes (Fig. 3b). In 4 of the 6 isolates, *bla*_KPC_ was harbored in a variant of NTE_KPC_ designated NTE_KPC_-IIe. Upstream of *bla*_KPC_ gene there was a 570 pb truncated *bla*_TEM-1_ gene (deletion of 291 pb) followed by an insertion sequence that was 84% similar to ISEc63, a 4473 bp element that belongs to the Tn3 family. This IS contains, a Tn3 family resolvase, a DDE-transposase (contains 3 acidic amino acids, DDE) and a 50 bp inverted repeat left of ISec63 (IRL, Fig. 3b). The DDE-transposase gene had a single nucleotide deletion of G384 that resulted in a frameshift from amino acid 128, producing a truncated protein in the 4 isolates. Downstream the *bla*_KPC_ gene there was the remnant of an ISKpn6 gene (ΔISKpn6, Fig. 3b), and the inverted repeat right of Tn4401 (IRR, Fig. 3b), traits that are common among most NTE_KPC_ sequences [10]. The ISec63-like element together with the *bla*_KPC_ gene plus Tn4401 remnants were inserted in the operon containing the genes required for conjugative transfer of the plasmid (TraI, TraD, VirB11, VirB9, VirB8, VirB5, and VirB4) (pink arrows, Fig. 3b). The region located upstream the ISec63-like element was highly variable among the 6 isolates analyzed: Eco-5, Eco-7, and Ecl-6 lack some genes respective to isolates Kpn-3, Ecl-9, and Eco-8. It could also be stated that Ecl-9 and Eco-8 are different from Kpn-3 (Fig. 3b).

No other IS or transposon structure was found in the plasmid. The plasmid contained other genes coding for proteins involved in antibiotic resistance, eg: Sul1 (sulphonamides), QnrB (quinolones), QacEΔ1 (quaternary ammonium compounds), AadA16 and AacA4 (aminoglycosides) (Fig. 3b). Additionally, it harbored genes involved in DNA repair and metabolism (UmuCD operon, DNA-cytosine methyltransferase, antirestriction protein KlcA and EcoRII).

## Discussion

Our results show that KPC-producing *P. aeruginosa* possessed the *bla*_KPC_ gene in a different plasmid and transposon structure from that found in *Enterobacteriaceae*, suggesting that there was no plasmid transfer between them as initially hypothesized.

The multispecies outbreak described herein was most likely driven by horizontal plasmid transfer among *Enterobacteriaceae* species. Indeed, all enterobacterial isolates studied with WGS harbored essentially the same *bla*_KPC_-bearing IncN plasmid. Importantly, this genetic element has been described as a conjugative free mobility plasmid between different species [22]. The fact that most enterobacterial isolates of the same species carrying the IncN plasmid were not clonal further supports the horizontal transfer mechanism.

NTEs have been classified based on the genes adjacent to *bla*_KPC_ gene: type NTE-I has no insertions respective to the first variant found in China, NTE-II has a partial *bla*_TEM_ gene upstream *bla*_KPC_, and NTE-III has an insertion of *tnpR* (Tn*5563*)/IS6100 [10]. The Argentinian and previous Chilean NTE_KPC_ variants were of the type NTE_KPC_-Ia and were identical to that firstly reported in China. A novel NTE_KPC_-IId element has been recently described in Brazil, in which there is a truncated *bla*_TEM_ gene upstream the *bla*_KPC_ gene, and a truncated ISKpn6 gene followed by *relE/parE* toxin-antitoxin system downstream the *bla*_KPC_ gene [16]. The NTE_KPC_-IIe described here is a novel variant based on the presence of an array of *vir* genes downstream the *bla*_KPC_ gene, a region that is usually less variable than the upstream region. A detailed genomic analysis done in Colombia with isolates obtained during KPC emergence showed that the first events responsible for KPC dissemination were horizontal transfer of mobile genetic elements carrying *bla*_KPC-2_ in the typical Tn4401 transposon and also in NTE_KPC_ elements, followed by introduction of *K. pneumoniae* ST258 carrying *bla*_KPC-3_ exclusively in Tn4401 and its subsequent clonal dissemination [9]. The events described in Argentina illustrate a similar picture: the first KPC-producing bacteria were of different enterobacterial species and non-ST258 *K. pneumoniae* carrying *bla*_KPC_ in NTEs, followed by introduction and clonal dissemination of *K. pneumoniae* ST258 carrying *bla*_KPC-3_ in a typical Tn4401 element [12]. The events described in this work are similar to the beginning of the Colombian and Argentinian KPC epidemics: the *bla*_KPC-2_ gene embedded in NTEs disseminates among various enterobacterial species and non-ST258 *K. pneumoniae*. It would be very interesting to analyze recent Chilean isolates to determine if the switch to the predominant ST258 carrying *bla*_KPC-3_ in Tn4401 has occurred.

One of the most remarkable limitations of this work is the lack of experimental evidence about the transferability of the IncN plasmid intra and interspecies. Additionally, the WGS method used provided short reads which make it difficult to obtain a circular complete sequence. This could explain the incomplete sequences obtained for some strains like Eco-5 and Ecl-6. Of note, enterobacterial isolates from patients P4 and P5 lost their KPC genes and were not sequenced. These isolates could have lost their KPC-bearing plasmids, maybe because these plasmids were different and less stable than those of isolates from patient P3, that were stably maintained. In fact, the band of less than 4 Kb of isolate Ecl-12 could correspond to a plasmid belonging to a different incompatibility group. It is possible that interspecies plasmid transfer occurred only in patient P3.

Based on our clonality analysis, only one case of intra-hospital transmission could have occurred: Pae-1 and Pae-13, isolated from P1 and P6 respectively; both isolates were clonal and both patients were hospitalized in the same service, although 2 months apart. Infection control measures such as hand-washing, sterilization of medical devices and contact isolation precautions might have failed in this particular case.

Isolates Pae-1 and Pae-13 were ST654, that is a high-risk clone and has been previously reported in Argentina [23] associated with production of KPC carbapenemase. Sequence types determined for *E. coli* and *C. freundii* isolates are not high-risk clones and no previous results were found about these STs associated with carbapenemase production. *E. cloacae* isolates were of the types ST45 and ST114, that have been previously described in other studies associated with the production of ESBLs and carbapenemases, with ST114 being a high-risk clone [24]. None of the *K. pneumoniae* isolates recovered belonged to the globally disseminated high-risk clone ST258; two of the isolates were ST11 and one was ST25. However, ST11 is also a clinically relevant genetic lineage, being closely related to ST258 [25]. Although ST25 is less frequent, it is associated with KPC production and it has been previously reported in Chile by the Institute of Public Health (unpublished data). Moreover, ST25 has been described as an hypervirulent clone associated with mucoid phenotype [26].

## Conclusions

We describe here a multispecies outbreak of KPC-producing bacteria driven by horizontal gene transfer of *bla*_KPC_ gene embedded in a novel NTE element, named NTE_KPC_-IIe. This type of transmission poses a greater challenge to infection control measures often directed against containment of clonal dissemination.

## Methods

### Clinical isolates

We selected 13 carbapenemase-producing clinical isolates obtained between May and July, 2015, from 6 different patients (P1 – P6) (Table 1). Patients had been admitted to the intensive care unit (ICU), step-down unit, cardiology, surgical and pediatric care units at the Clinical Hospital of Red de Salud UC-CHRISTUS. Isolates species corresponded to *K. pneumoniae* (Kpn-3, Kpn-4, and Kpn-10), *Enterobacter cloacae* (Ecl-6, Ecl-9, and Ecl-12), *Escherichia coli* (Eco-5, Eco-7, and Eco-8), *Citrobacter freundii* (Cfr-11) and *P. aeruginosa* (Pae-1, Pae-2, and Pae-13). All included isolates were obtained from surveillance cultures (rectal swabs), as part of an institutional protocol designed to actively screen for carbapenemase-producing bacteria. Such protocol was instituted in 2013 and consisted of monthly rectal swabs performed to all patients hospitalized for more than 5 days in any of the intensive care units of the hospital [27]. Importantly, despite finding many carbapenem-resistant organisms, no carbapenemase-producing bacteria had been recovered before the outbreak described herein [27]. Swabs were plated in chromogenic medium ChromID CARBA (bioMèrieux). Colonies were transferred to Mueller-Hinton agar plates and bacterial species was determined with Matrix-Assisted Laser Desorption/Ionization - Time of Flight (MALDI-TOF) (Bruker-Daltonics, Germany). Carbapenemase production was assessed through the Carba-NP test performed according to CLSI instructions [28].

### Detection of carbapenemases genes

Total DNA extraction was performed using the Magna Pure Compact Nucleic acid isolation kit I kit (Roche). The presence of *bla*_KPC_ and *bla*_VIM_ genes was determined through PCR using primers listed in Table 3 as previously described [29]. PCR products were purified and sequenced bi-directionally (Macrogen Inc., Korea). The primers used to sequence *bla*_KPC_ and *bla*_VIM_ genes are provided in Table 3 [11, 30]. All sequences were corrected and analyzed using Chromas Lite and Clustal Omega online software. To determine the type of KPC and VIM carbapenemases, sequences were compared to the GenBank online database using BLAST software (Basic Local Alignement Search Tool).

**Table 3:** Primers used in this study

Gene	Primers	Sequence	PCR product size (bp)
*bla*_KPC_	KPC-F KPC-R	5′-TGTCACTGTATCGCCGTC-3′ 5′-CTCAGTGCTCTACAGAAAACC-3′	1010
*bla*_VIM_	VIM-F VIM-R	5′-CCGATGGTGTTTGGTCGCAT-3′ 5′-GAATGCGCAGCACCAGGAT-3`	391
*bla*_KPC_	KPC-Up KPC-Dw	5′-GCTACACCTAGCTCCACCTTC-3′ 5′-ACAGTGGTTGGTAATCCATGC-3’	968
*bla*_VIM_	VIM-Up VIM-Dw	5’-ATTGGTCTATTTGACCGCGTC-3′ 5′-TGCTACTCAACGACTGAGCG-3’	780

Primers KPC and VIM -F and -R were used for PCR gene detection

Primers KPC and VIM -Up and -Dw were used for gene sequencing

### Pulsed Field Gel Electrophoresis (PFGE)

Pulsed-field gel electrophoresis (PFGE) of genomic DNA macrorestricted with SpeI or XbaI enzymes was performed to establish a clonal relationship between isolates of the same species, according to the PulseNet protocol of CDC [31]. PFGE conditions were as follows: pulse times ranged from 2 s to 40s for 18 h at 6.0 V/cm at 14 °C. The PFGE profiles obtained were analyzed with GelJ 2.0 software [32] and a dendrogram was constructed using the Dice coefficient (Tolerance 2%) and unweighted pair group method with arithmetic mean (UPGMA). The similarity between band patterns was interpreted according to Tenover criteria [33] setting 95% similarity cut-off values for identifying pulsotypes.

### Multi Locus Sequence Typing (MLST)

Amplification and sequencing of 7 housekeeping genes were performed according to Pasteur Institute [34] or PubMLST [35] protocols, and PCR products were sequenced bidirectionally (Macrogen Inc., Korea). Subsequently, the sequences were analyzed using ClustalW Omega online software. The electropherograms were analyzed using Chromas Lite software. Corrected sequences were uploaded to the MLST database to obtain sequence types based on allele combinations.

### Plasmid analysis

Identification of plasmid incompatibility groups was performed by multiplex PCR, recognizing incompatibility groups from *Enterobacteriaceae* and *P. aeruginosa* as previously described [19, 20].

For plasmid extraction, a single colony of an overnight culture in blood agar plates was resuspended in 2 ml microcentrifuge tubes containing 500 μL distilled water and centrifuged at 3100 g for 10 min. Bacterial pellets were resuspended in 400 μL of cold solution I containing 2 mg/mL lysozyme, 50 mM glucose, 10 mM EDTA, 25 mM Tris-HCl and 0.2 mg/mL RNAse. Samples were stirred vigorously and left at room temperature for 5 min. Eight hundred μL of solution II containing Sodium Hydroxide / Sodium dodecyl sulfate was added, mixed 3 times by inversion, and incubated in ice for 20 min. Six hundred μL of solution III (3 M Sodium Acetate, pH 5.2) was added, vortexed vigorously and incubated for 5 min in ice and then centrifuged at 12,400 g for 15 min. The supernatant (500 μL approx.) was transferred to another tube and mixed with an equal volume of Phenol: Chloroform: Isoamyl alcohol (25: 24: 1), the mixture was centrifuged at 12,400 g for 10 min. Supernatants (500 μL approx.) were transferred to another tube and 2 volumes of cold absolute ethanol were added; it was gently stirred for 2 min and incubated for 35 min at room temperature and centrifuged at 12,400 g for 20 min. Absolute ethanol was removed and 1 volume of cold 70% ethanol was added, subsequently, it was incubated for 5 min and centrifuged for another 5 min at 12,400 g. Finally, ethanol was removed, and the DNA pellet allowed to dry at room temperature. DNA was resuspended in TE (Tris-EDTA) buffer pH 8.0 and stored at − 20 °C. Plasmid DNA was separated on a 0.75% agarose gel electrophoresis for 5 h at 50 V.

### Library preparation and DNA sequencing

Unfortunately, when isolates were regrown for genomic DNA extraction and whole-genome sequencing (WGS) isolates from P4 and P5 had lost the plasmid harboring the *bla*_KPC_ gene (Kpn-10, Cfr-11, and Ecl-12). Isolate Kpn-4 did not lose its KPC gene but its WGS data were low quality, thus it was not included. For this reason, only WGS data of 7 of the 13 original isolates were obtained, and it was performed by Novogene (California, US). A 350 bp insert DNA library was prepared and sequencing was performed in an Illumina Platform PE150. The Q30 obtained was > 90% for all 7 isolates.

### Read processing, de novo assembly and annotation of plasmid genomes

Reads were adapter trimmed using Trimmomatic 0.30 with a sliding window quality cutoff of Q15. De novo assembly was performed on samples using plasmidSPAdes as part of the core SPADES version 3.7 package [36]. Genomic annotation of the recovered draft genomes was performed with Prokka tool 1.11 [37]. Annotations were manually reviewed using BLASTP+ against the non-redundant protein NCBI database. Contigs were further aligned against plasmid pEC881_KPC (accession number #CP019026.1) for *Enterobacteriaceae* and plasmid pPA2047 (accession number MN082782) for *P. aeruginosa*. Contigs were scaffolded using the MeDuSa open software [38].

### Synteny and comparative genomic analyses

Comparisons between individual genomes were performed using BLASTn. Identification of insertions, deletions, and variations in syntenic regions was performed using Easyfig v2.1 [39] using the BLASTn comparison file and gbk files as inputs and calibrating the tBLASTx identity values to a minimum 99% ID for the reference plasmids used. Final visualization and annotations of the aligned contigs ORFs (Open Reading Frames) were made using Geneious vR.10.

## Supplementary Information

**Additional file 1: Figure S1.** Unproccessed Pfge Gels.

## Data Availability

The datasets used and/or analysed during the current study are available from the corresponding author on reasonable request. All data generated or analysed during this study are included in this published article (and its supplementary information files). Genbank accession numbers for deposited data are as follows: MT949189 for Kpn-3; MT949193 for Ecl-6, Ecl-9 and Eco-5; MT949190 for Eco-8; and MT949192 for Eco-7.

## Abbreviations

ATCC: American Type Culture Collection
CG: Clonal group
CLSI: Clinical and Laboratory Standards Institute
EDTA: Ethylenediaminetetraacetic acid
ICU: Intensive Care Unit
IRL: Inverrted Repeat Left
IRR: Inverted Repeat Right
MALDI-TOF: Matrix Assisted Laser Desorption - Time of Flight Mass Spectrometry
MLST: Multi-Locus Sequence Typing
NTE: Non-Tn4401 Element
ORF: Open Reading Frame
PFGE: Pulsed-field gel electrophoresis
ST: Strain Type
UPGMA: Unweighted Pair Group Method with Arithmetic mean
WGS: Whole Genome Sequencing
WT: Wild type
