# Supplementary figures and images for: A multispecies outbreak of carbapenem-resistant bacteria harboring the blaKPC gene in a non-classical transposon element
Source: BMC Microbiol. 2021 Apr 9;21:107. doi: 10.1186/s12866-021-02169-3 (PMC8034096; doi:10.1186/s12866-021-02169-3)

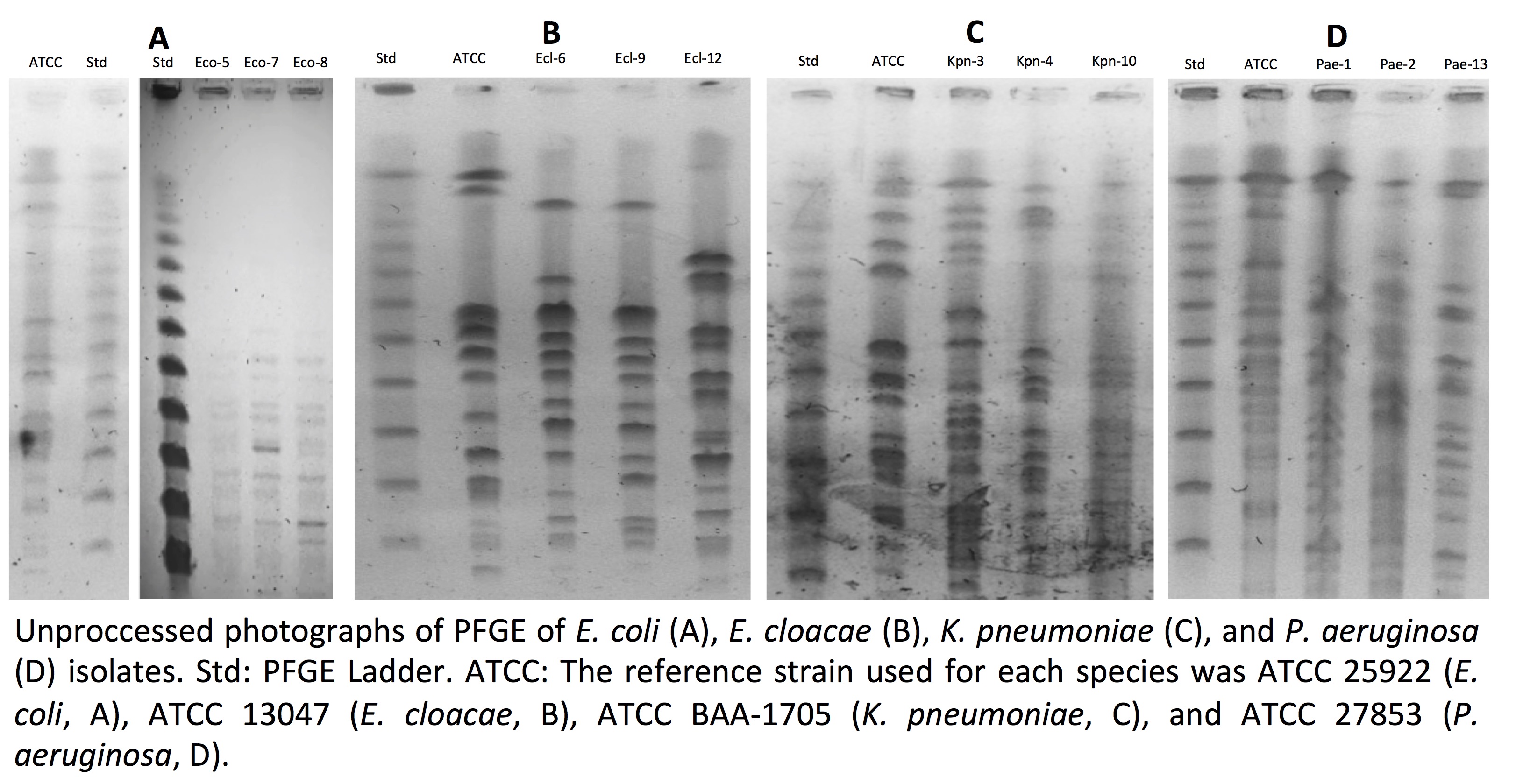

Supplement: Supplementary file 1 — Additional file 1: Figure S1. Unproccessed Pfge Gels. [file 12866_2021_2169_MOESM1_ESM.jpg]
